# *Intraoperative* but not *postoperative* blood transfusion adversely affect cancer recurrence and survival following nephrectomy for renal cell carcinoma

**DOI:** 10.1038/s41598-018-37691-4

**Published:** 2019-02-04

**Authors:** Yasmin Abu-Ghanem, Zohar Dotan, Dorit E. Zilberman, Issac Kaver, Jacob Ramon

**Affiliations:** 1 Department of Urology, Sheba Medical Center, Tel Hashomer, Israel; 2 Sackler Faculty of Medicine, Tel Aviv University, Tel Aviv, Israel

## Abstract

The association between perioperative blood transfusion (PBT) with adverse oncological outcomes have been previously reported in multiple malignancies including RCC. Nevertheless, the importance of transfusion timing is still unclear. The primary purpose of this study is to appraise whether the receipt of intraoperative blood transfusion (BT) differ from postoperative BT in regards to cancer outcomes in renal cell carcinoma (RCC) patients treated with nephrectomy. Data on 1168 patients with RCC, who underwent radical or partial nephrectomy as primary therapy between 1988–2013 were analyzed. PBT was defined as transfusion of allogeneic red blood cells (RBC) during surgery or the postsurgical period. Survival was analyzed and compared using the Kaplan–Meier method with the log-rank test. Of 1168 patients, 198 patients (16.9%) received a PBT. Including 117 intraoperative BT and 81 postoperative BT. Only 21 (10.6%) patients required both intraoperative and postoperative BT. On multivariate analyses, receipt of PBT was associated with significantly worse local disease recurrence (HR: 2.4; P = 0.017), metastatic progression (HR: 2.7; P = 0.005), cancer-specific mortality (HR: 3.5; P = 0.002) and all-cause mortality (HR: 2.1; P = 0.005). Nevertheless, postoperative BT was *not* independently associated with increased risk of local recurrence (p = 0.1), metastatic progression (P = 0.16) or kidney cancer death (P = 0.63), yet did significantly increase the risk of overall mortality (HR: 2.6; P = 0.004). In the current study, intraoperative transfusion of allogeneic RBC is associated with increased risks of cancer recurrence and mortality following nephrectomy.

## Introduction

The association between perioperative blood transfusion (PBT) and adverse cancer-specific outcomes following cancer surgery has been documented in several malignancies including colorectal, lung and bladder cancer^1–3^. For kidney cancer, although the literature has conflicting reports, most recent studies have identified that PBT is associated with negative outcomes in the setting of nephrectomy for renal cell carcinoma (RCC)^4–6^. Despite existing data, in the last decade, the deleterious effects of blood transfusion on prognosis continue to be investigated and are considered to be of increasing clinical importance. Concurrently, the immunosuppressive effect from BT is being explored, and additional mechanisms are being suggested to explain this association. Interestingly, some of the proposed mechanisms, including immune function impairment from anesthetic agents^7^, or decreased host immunity caused by tissue injury are likely to have an added effect during surgery^8^. Hence, intraoperative transfusion may potentially have a more significant effect on patients’ outcomes. In support of this idea, recent studies by Abel E. *et al*.^9^ and Moschini M. *et al*.^10^ have addressed this issue in bladder cancer surgery patients and demonstrated that only intraoperative BT was associated with poor recurrence and cancer-specific outcomes. However, the results are still preliminary, and there is still a paucity of data regarding the timing of transfusion and its effect on oncological outcomes in other malignancies. The aim of the current study is to assess the association of intraoperative and postoperative blood transfusion with cancer-related outcomes in patients undergoing nephrectomy for RCC.

## Patients and Methods

We identified all patients who underwent elective partial or radical nephrectomy at our institute between 1988 and 2013. Patients’ clinicopathological variables were collected retrospectively, following approval is given by the Sheba Medical Center Institutional Review Board (IRB)/Ethics (Helsinki) Committee, in accordance with relevant guidelines and regulations. Our IRB waived the need for informed consent. Clinicopathologic variables reviewed included: age, gender, Body Mass Index (BMI), American Society of Anesthesiologists physical status classification (ASA) score, symptoms, preoperative hemoglobin, type of nephrectomy, estimated surgical blood loss (EBL) receipt of PBT (intra and post-operative) and number of units transfused; type of nephrectomy (Radical vs. Partial) and surgical approach (Laparoscopic vs. Open; robotic partial nephrectomies were excluded from the current study); pathologic T and N stages (tumor stage coded as pT2 or less and pT3), nuclear grade, tumor necrosis, presence sarcomatoid differentiation and capsular invasion, presence of positive tumor margins in final pathology (PSM) and tumor location (central). Exclusion criteria included: patients who received adjuvant treatment, patients with additional tumors (other than RCC), benign histology and metastatic disease upon diagnosis (M1). Perioperative blood transfusion was defined as transfusion of allogeneic red blood cells (RBC) during surgery or the postsurgical period. The technique of open NSS is as described in previous publications. We used a vascular clamp for hilar clamping in all cases^11–13^. Notably, all patients with symptomatic anemia regardless of hemoglobin level or patients with coronary artery disease and hemoglobin levels below 10 g/dL received PBT. Transfusion with other blood products was not recorded. The TNM staging system has been used for disease staging. Structured preoperative staging and follow-up routine were conducted according to accepted European Urology Association guidelines (EAU) for RCC^14^. In general, follow-up was conducted semi-annually for the first 5 years after surgery (including chest, abdomen and pelvis imaging) and annually thereafter. All patients with post-operative follow- up of fewer than 6 months were excluded from the current study. We used Kaplan-Meier method with a log-rank test to evaluate the rates of recurrence-free survival (RFS; local and distant), cancer-specific survival (CSS) and overall survival (OS). Cox proportional hazards regression model was used to evaluate the relations between the timing of PBT and outcome, controlling for clinicopathologic parameters. A p value of less than 0.05 was considered to be indicative of statistical significance. Statistical Package for Social Sciences (SPSS, Version 22.0, Chicago, IL, USA) was used for all analyses.

## Results

Overall 198 of 1168 (16.9%) received a PBT with a median (IQR) number of units transfused of 2 units (1–3) (Table 1). Of these, 138 (69.7%) received an intraoperative BT, and 81 patients (40.9%) received a postoperative BT. More specifically, 117 patients received intraoperative BT alone, 60 patients received postoperative BT alone and only 21 (10.6%) of the patients who were transfused intra-operatively required additional postoperative transfusions. Given the small number of patients, this sub-group was excluded from further analysis. Median (IQR) follow-up after surgery was 63 months (32–102). One hundred and sixty-five (14%) patients had disease recurrence, of which 77 had local recurrence (LR), and 88 had distant (metastatic) progression (MP). Two hundred and fifty-five patients died during follow up. In 55, the cause of death was RCC.

**Table 1:** Univariate analysis of predictive factors for perioperative blood transfusion among patients undergoing Nephrectomy for renal masses.

Variable	Total (N = 1168)	Intra-operative BT (n = 117)	Post-operative BT (n = 60)	No PBT (n = 982)	*p*-value
Age, yr, median (IQR)	64 (55–72)	66 (62–68)	67 (63–68)	63 (54–71)	0.5
Gender, no. (%)					0.02
Male	744 (63.7)	64 (54.7)	34 (56.7)	635 (66.1)	
Female	415 (36.3)	53 (45.3)	26 (43.3)	326 (33.9)	
Operation					0.08
PN	582 (49.8)	46 (39.3)	36 (60)	500 (50.9)	
RN	586 (50.2)	71 (60.7)	24 (40)	482 (49.1)	
Tumor size, cm median (IQR)	4 (2.8–6)	5.7 (3–7)	4.7 (3–6)	4.7 (3–7)	0.06
Central tumor (%)	287 (24.6)	40 (34.2)	22 (36.7)	225 (23.4)	0.004
Operation type					0.02
Open	722 (61.8)	90 (77)	36 (60)	578 (60)	
Laparoscopic	446 (38.2)	27 (23)	24 (40)	383 (40)	
preoperative Hb, g/dL	13.5 (12.4–14.6)	12.6 (12.4–14)	12.3 (12.1–14)	12.6 (11.3–13.6)	0.5
Nuclear grade, no.					
(%)**					0.07
1	105 (9.1)	10 (9.9)	5 (8.9)	80 (12)	
2	515 (44.4)	41 (40.6)	33 (58.9)	434 (59.2)	
3	252 (21.7)	36 (35.6)	16 (28.6)	193 (26.3)	
4	37 (3.2)	14 (13.9)	2 (3.6)	18 (2.5)	
Pathologic stage,					0.1
no. (%)					
≤T2	898 (76.9)	81 (69)	47 (78)	755 (78.6)	
≥T3	270 (23.1)	36 (31)	13 (22)	206 (21.4)	
Tumor necrosis no. (%)	215 (18.4)	32 (27.4)	16 (26.7)	156 (16.2)	0.9
Capsular invasion no. (%)	137 (11.7)	27 (23)	9 (15)	94 (9.8)	0.001
Sarcomatoid differentiation no. (%)**	21 (1.8)	4 (3.4)	3 (4)	14 (1.4)	0.001
PSM (%)	38 (3.2)	5 (4.4)	2 (3.3)	31 (3.3)	0.8
Symptoms no. (%)	342 (29.3)	53 (47.7)	25 (42.4)		0.5

**Missing data; abbreviation: PBT- peri-operative blood transfusion; BT- blood transfusion; PN- partial nephrectomy; RN- radical nephrectomy; Hb- Hemoglobin; PSM- positive surgical margins.

In the entire cohort, PBT was associated with poor outcomes including 5-yr RFS (81% vs. 92% P < 0.01), metastatic free survival (79% vs. 93% P < 0.001), CSS (85% vs 95%; P < 0.001) and OS (73% vs 81%; P < 0.001), respectively^15^. After stratification according to the timing of BT administration (postoperative transfusion vs. intraoperative transfusion), we compared the 3 groups of interest: Intraoperative vs. No BT, postoperative vs. No BT, Intraoperative vs. postoperative BT.

### Survival Estimates

**Figure 1:**
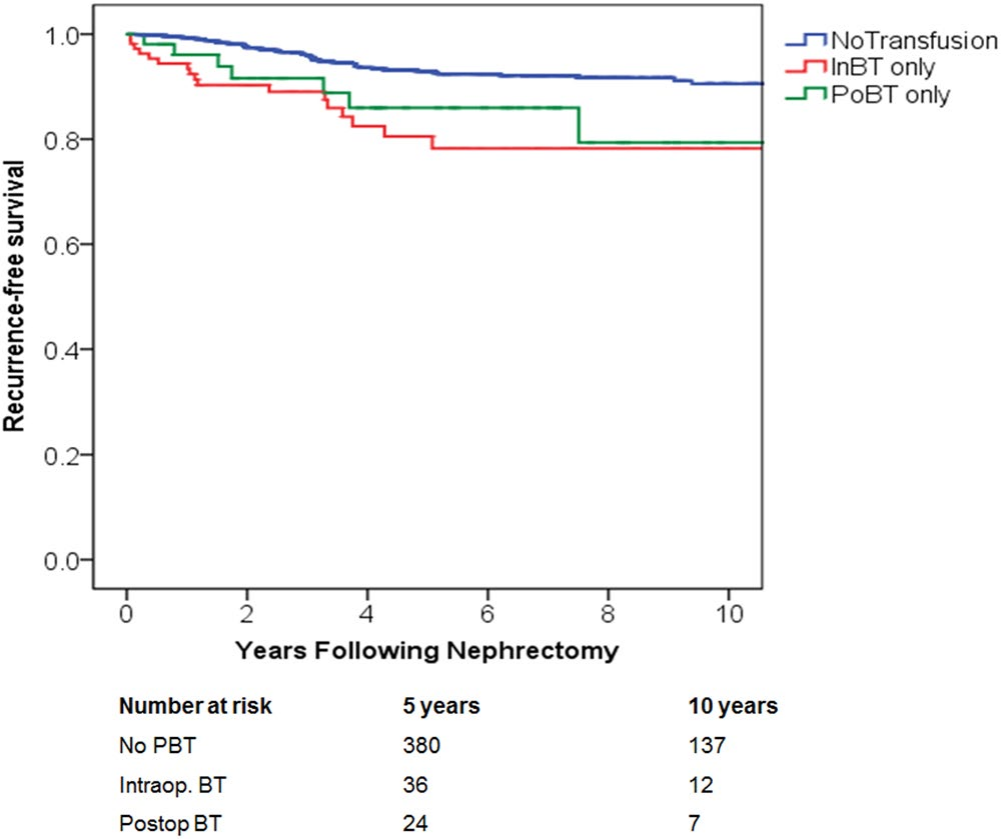
Recurrence-free survival (p < 0.001) among patients with renal cancer treated with nephrectomy, stratified by timing of perioperative blood transfusion. IntBT = intraoperative; blood transfusion, PoBT = postoperative blood transfusion.

**Figure 2:**
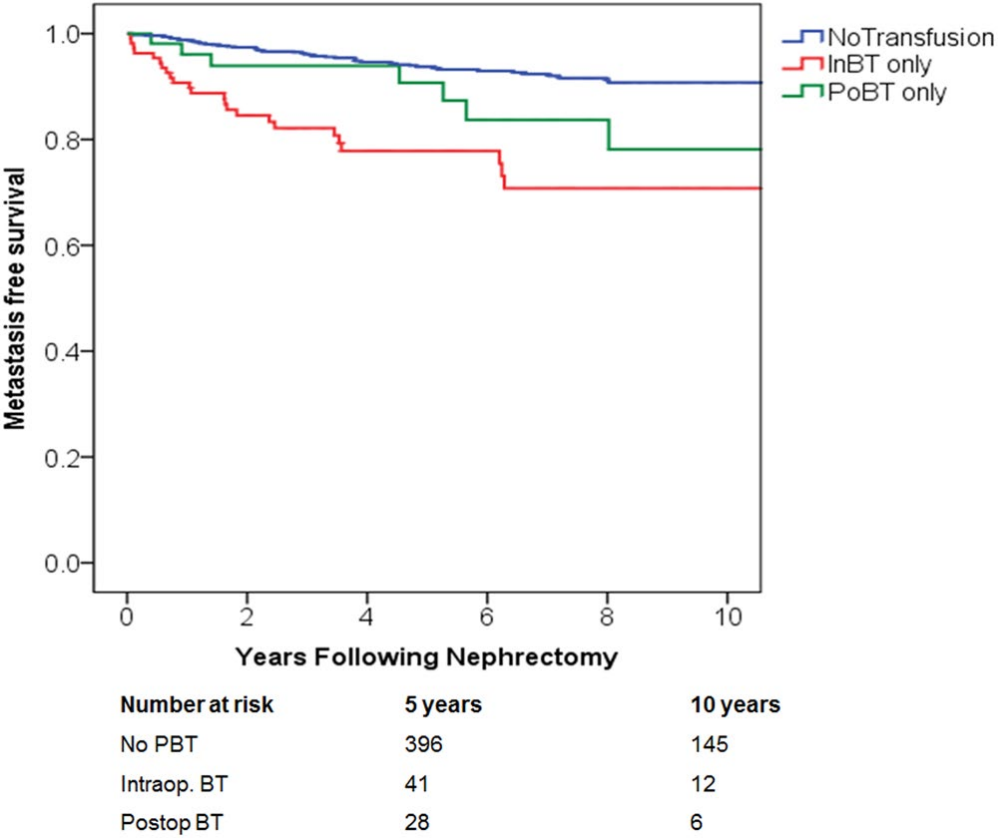
Metastasis-free survival (p < 0.001) among patients with renal cancer treated with nephrectomy, stratified by timing of perioperative blood transfusion. IntBT = intraoperative; blood transfusion, PoBT = postoperative blood transfusion.

Direct comparison revealed that intraoperative BT, but not postoperative BT is associated with poorer 5-year RFS when compared to those who were not transfused (78% vs. 92%; P < 0.01 and 86% vs. 92%; P = NS, accordingly), (Fig. 1). Similarly, the estimated 5-yr MFS was also significantly decreased among patients who received intraoperative BT compared to no PBT (78% vs. 93%), (P < 0.001). However, postoperative BT did not significantly confer the rate of MFS when compared to the no PBT group (84% vs. 93%, accordingly, P = NS), (Fig. 2).

**Figure 3:**
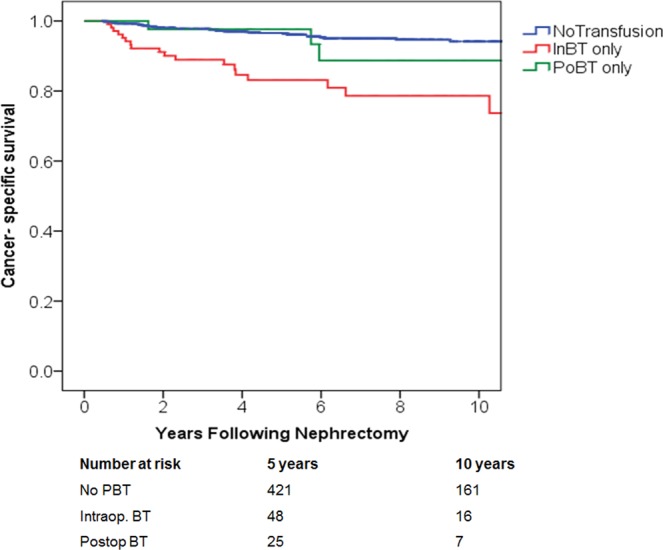
Cancer-specific survival (p < 0.001) among patients with renal cancer treated with nephrectomy, stratified by timing of perioperative blood transfusion. IntBT = intraoperative; blood transfusion, PoBT = postoperative blood transfusion.

In regards to CSS; intraoperative BT, but not postoperative BT was associated with poorer 5-year CSS when compared to those who were not transfused (84% vs. 95%; P < 0.001 and 92% vs. 95%; P = NS, accordingly), (Fig. 3). The association between timing of PBT and oncological outcomes were next tested using multivariate analysis (MVA) (Table 2). On MVA, intraoperative BT remained significantly associated with poorer outcomes including worse LR (HR: 2.3; P < 0.05), MP (HR: 2.2; P < 0.01), CSS (HR: 2.95; P < 0.01) and OS (HR: 2.0; P < 0.01). Postoperative BT did not significantly affect the rate of either LR (P = 0.1), MP (P = 0.1) or CSS (P = 0.5). However, it significantly increases the risk of overall mortality (HR: 2.6; P < 0.01).

**Table 2:** Multivariable Cox regression analyses predicting disease recurrence, cancer-specific mortality and any-cause mortality.

Variable	Local recurrence			Metastatic progression			Disease-specific mortality			All-cause mortality		
	HR	95% CI	P value	HR	95% CI	P value	HR	95% CI	P value	HR	95% CI	P value
Age (years)	0.99	0.97–1.02	**0.75**	0.98	0.96–1.0	**0.04**	1	**0**.**97**–**1**.**03**	**0.82**	1.03	1.01–1.05	**0.001**
Gender	0.6	0.3–1.15	**0.1**	0.5	0.25–1.0	**0.05**	0.5	0.2–1.19	**0.1**	0.8	0.5–1.3	**0.4**
ASA score	1.6	0.7–3.7	**0.25**	0.86	0.25–2.9	**0.8**	0.7	0.2–3.1	**0.65**	1.6	0.8–2.8	**0.1**
Tumor Size	0.99	0.89–1.1	**0.95**	1.07	0.98–1.17	**0.1**	1.09	0.96–1.2	**0.2**	1.04	0.96–1.1	**0.3**
preoperative Hb, g/dL	0.97	0.8–1.2	**0.8**	0.89	0.7–1.1	**0.3**	0.96	0.75–1.2	**0.7**	1.01	0.86–1.2	**0.9**
PBT												
Intraoprative BT	2.4	1.2–4.8	**0.02**	2.77	1.36–5.6	**0.005**	3.53	1.6–7.7	**0.002**	2.1	1.26–3.5	**0.005**
Postoprative BT	2	0.8–4.8	**0.1**	1.89	0.8–4.7	**0.2**	1.37	0.38–4.9	**0.6**	2.4	1.3–4.4	**0.004**
Nuclear Grade	1.6	1.1–2.4	**0.02**	1.42	0.95–2.1	**0.09**	1.11	0.7–1.8	**0.7**	1.03	0.76–1.4	**0.8**
Tumor Stage (pT3–4 compared with pT1-T2)	2.14	1.02–4.5	**0.045**	4.69	2.3–9.5	**0.001**	2.42	1.06–5.6	**0.04**	2.16	1.3–3.7	**0.005**
Tumor necrosis	1.06	0.5–2.05	**0.9**	1.88	0.99–3.5	**0.05**	2.65	1.26–5.6	**0.01**	1.73	1.1–2.77	**0.02**
Capsular invasion	0.61	0.25–1.5	**0.3**	0.6	2.7–1.3	**0.2**	1.11	0.45–2.7	**0.8**	0.96	0.5–1.7	**0.9**
Symptoms	1.11	0.6–2.02	**0.7**	1.11	0.56–2.2	**0.8**	0.93	0.42–2.1	**0.9**	1.24	0.77–1.98	**0.4**
Year of surgery	0.99	0.9–1.1	**0.95**	0.98	0.91–1.05	**0.6**	0.99	0.9–1.1	**0.9**	0.91	0.86–0.97	**0.002**

Abbreviation: PBT- peri-operative blood transfusion; BT- blood transfusion; Hb- Hemoglobin.

## Discussion

In the current study, we found that receipt of intraoperative BT, but not post-operative BT, was associated with adverse oncological outcomes in patients treated with nephrectomy for RCC. Previous studies investigating the impact of PBT on survival in several malignancies including colon, bladder, pancreatic and hepatic cancers^6,11,12^. In most studies to date, appropriate timing and use of blood transfusion in the operating room are poorly defined, leading to a paucity of data regarding the timing of transfusion and its association with disease recurrence and mortality. Recently, Abel E. *et al*.^9^, assessed the question of timing and examined the potential association between intraoperative BT and oncological outcomes in patients undergoing radical cystectomy (RC) for bladder cancer. Study results showed that intra-operative BT, but not postoperative transfusion, was associated with poorer risk of disease recurrence and kidney cancer death. Few recent studies by Moschini M. *et al*.^10,14,15^ confirmed these results, also on RC patients (Table 3).

**Table 3:** Published results Timing of blood transfusion.

Study	Operation	Year	N	% PBT		*Survival analysis (HR, 95% CI)*					
				Intra-OP	Post-OP	Intra-OP			Post-OP		
Abel E, *et al*.	RC	2014	360	18	22	Not Sig.	**Significant 1.8 (1.1–2.9)**	Not Sig.	Not Sig.	Not Sig.	Not Sig.
			1770	23.4	16.1	**Significant 1.45 (1.2–1.8)**	**Significant 1.55 (1.2–1.9)**	**Significant 1.4 (1.2–1.6)**	Not Sig.	Not Sig.	Not Sig.
Moschini M, *et al*.	RC	2015	1490	21.6	6.5	**Significant 1.2 (1.03–1.6)**	**Significant 1.6 (1.2–2.3)**	**Significant 1.45 (1.02–2.1)**	Not Sig.	Not Sig.	Not Sig.
Moschini M, *et al*.	RC	2016	728	N/A	N/A	**Significant 1.4 (1.1–1.97)**	**Significant 1.6 (1.1–2.3)**	**Significant 1.5 (1.1–2.1)**	Not Sig.	Not Sig.	Not Sig.
Moschini M, *et al*.	RC	2017	1081	11.3	7	^ψ^ Not Sig.	N/A	N/A	^ψ^ Not Sig.	N/A	N/A
			433	28.2	6.5	^ψ^ Not Sig.	N/A	N/A	^ψ^ Not Sig.	N/A	N/A
Current study	PN, RN		1168	11.8	6.9	**Significant 2.4 (1.2–4.8)**	**Significant 3.5 (1.6–7.7)**	**Significant 2.1 (1.3–3.5)**	Not Sig.	Not Sig.	**Significant 2.4 (1.3–4.4)**

Abbreviation: RC- radical cystectomy; PN- partial nephrectomy; RN- radical nephrectomy; PBT- peri-operative blood transfusion; Intra-op- intra operative; Post-op- post operative; Sig.- significant; N/A- not available.

The present data provide further support for these findings, yet, in RCC patients. In the current cohort, patients receiving an intraoperative transfusion had a greater than twofold risk of developing perioperative complications including local recurrence, metastatic progression, and mortality from any cause and RCC. However, patients who received postoperative transfusion had increased risk for overall mortality but not CSS or disease recurrence or progression. There are a few potential ways to explain these results. First, although the two groups of patients (intra and post-operative BT) were comparable in all clinic-pathologic characteristics, administration of intra-operative BT may, in part, be secondary to increased surgical complexity or to measures of increased patient frailty which may not only reflect clinicians’ decisions regarding BT but also the patients general condition and their survival chances. Since the decision of BT delivery during the postoperative course also depends on the physician’s judgment and view of the patient, this could also explain why post-operative BT delivery was found associated with worse OS. However, the fact that post-operative BT was not related to adverse cancer-related outcomes may suggest that the association between intraoperative BT and prognosis is independent and specific. Second, different sample sizes; and third, the potentially more escalated immunosuppressive effect from BT delivered intraoperatively. So far, the association of PBT with worse cancer outcomes continues to be controversial. The mechanism underlying the adverse effects of blood transfusion has been assumed to be related to the suppressive effects on the immune system. Several studies have reported that blood transfusion suppresses host immune system via a reduction in T lymphocytes function, inhibition of interleukin-2, increased numbers of T-suppressor cells and release of immunosuppressive prostaglandins^3,16–19^. Other mechanisms suggested, include reduced host defense caused by opioids, local anesthetics, ketamine, and non-steroidal anti-inflammatory drugs^7^ or by tumor cells spread due to invasive intervention^8^. It is possible that the transient immune impairment induced by certain anesthetic agents or surgical manipulation allied to the blood transfusion may create an additive or synergistic immunosuppressive condition favorable to cancer cell survival and spread in the perioperative period^9^. These suggested mechanisms may have a paramount effect during operation and thus lead to poorer outcomes in patients who receive blood products at this point as opposed to the post-operative period. Moreover, recent findings suggest an association between intraoperative transfusion and perioperative morbidity and mortality. Bernard *et al*. for example^20^, found that the use of even 1 or 2 units of blood during general surgical operations was associated with increased perioperative morbidity and mortality. Comparable results were also found in patients who required transfusion during various thoracic^18^ or vascular^19^ operations. Such findings, support the proposed effect of BT, particularly intraoperatively. It is not debatable that blood transfusion may be necessary and even life-saving in many situations, especially in the intraoperative setting. However, looking at the current data, it could be safety suggested that not *all* BT administrated during the operation were lifesaving or even necessary. In the present cohort, more than one-half (59%) of the patients transfused during surgery did not require any additional postoperative transfusion. Moreover, the majority of patients transfused intraoperatively received only 1 unit (42%) or 2 units (34%) of blood, and most of these patients (89%) did not need any additional blood products after surgery. This inconsistency may be related to the different factors that influence decision- making between the surgeon and the anesthesiologist as well as the lack of definitive guidelines for *intraoperative* transfusion. Despite existing data on the optimal timing and use of BT, significant variability still exists. In fact, Frank *et al*. previously identified the considerable variation among surgical services and procedures, and among individual anesthesiologists and surgeons regarding the decision to give perioperative blood transfusion^21^. These findings highlight the necessity for evidence-based protocols for blood administration during both the intraoperative and perioperative period. In order to relegate the potential overuse of blood products, further studies are necessary to examine variation in hemoglobin triggers and overall utilization of intraoperative blood transfusions. Study limitations include the retrospective nature of this study, and the long duration (1988–2013). Surgical treatment for renal masses and overall utilization of transfusion has changed considerably during that timeframe and may insert additional variability into the data. One way to rout this problem is by including the variable “year of surgery” in the Cox proportional hazard regression model (Table 2). As shown in the table, as expected, experience, and maybe improved techniques may have affected the overall survival rate^22–25^, but not the disease-specific survival, that was significantly associated with PBT.

## Conclusions

Intraoperative blood transfusion is associated with increased risk of disease recurrence and cancer-specific mortality. The current data further underline the potential adverse outcomes associated with PBT and the need for more restrictive transfusion criteria to minimize transfusion rate, especially at the intraoperative setting.
